# Barriers and facilitators to implement the redispensing of unused oral anticancer drugs in clinical care: A hybrid-effectiveness type I study

**DOI:** 10.1016/j.rcsop.2024.100493

**Published:** 2024-08-17

**Authors:** Elisabeth M. Smale, Eva W. Verkerk, Eibert R. Heerdink, Toine C.G. Egberts, Bart J.F. van den Bemt, Charlotte L. Bekker

**Affiliations:** a Radboud University Medical Center, Department of Pharmacy, Nijmegen, the Netherlands; b Radboud University Medical Center, Department of IQ Healthcare, Nijmegen, the Netherlands; c University Medical Centre Utrecht, Division of Laboratory, Genetics and Pharmacy, Department of Clinical Pharmacy, Utrecht, the Netherlands; d Utrecht University, Faculty of Science, Utrecht Institute for Pharmaceutical Sciences, Division of Pharmacoepidemiology and Clinical Pharmacology, Utrecht, the Netherlands; e Utrecht University of Applied Medical Centre Utrecht, Research Group Innovations of Pharmaceutical Care, Utrecht, the Netherlands; f Sint Maartenskliniek, Department of Pharmacy, Ubbergen, the Netherlands

**Keywords:** Medication waste, Medication redispensing, Oral anticancer drugs, Stakeholder engagement, Hybrid effectiveness-implementation design, Qualitative research

## Abstract

**Background:**

Minimizing medication waste through the redispensing of oral anticancer drugs (OADs) that were unused by patients provides economic and environmental benefits, but this is not yet universally implemented in clinical care.

**Objective(S):**

To identify barriers and facilitators to the implementation of redispensing unused OADs in clinical care.

**Methods:**

A multicentre intervention study following a hybrid effectiveness-implementation type I design was conducted, consisting of semi-structured interviews with key stakeholders involved in the redispensing program: pharmacy employees, prescribing clinicians in oncology and haematology, patients who participated in redispensing and patients who declined trial participation. Questions encompassed experiences and suggestions for future implementation. The Consolidated Framework for Implementation Research (CFIR) guided data collection and categorisation of identified barriers and facilitators through thematic analysis.

**Results:**

In total, 35 interviews were conducted, identifying 15 themes encompassing barriers and facilitators, reflecting all CFIR domains. Facilitators encompassed: 1) convenient process requiring an acceptable time-investment; 2) support from project leaders and implementation champions; 3) being well-motivated by personal values and societal impact; 4) feeling ensured of medication quality upon redispensing; 5) endorsement by healthcare providers for patient participation; 6) clear and personal patient communication; 7) good visibility of intervention successes; and 8) implementation well supported through a collaborative network. Barriers encompassed: 1) unclear target population; 2) redispensing legally prohibited; 3) absence of financial compensation for pharmacies; 4) complexity arising from two parallel work processes; 5) widespread communication on adjustments within local teams challenging; 6) patient's low receptiveness due to burden of oncology treatment; and 7) lack of familiarization among pharmacy technicians.

**Conclusions:**

Facilitators for implementation of redispensing unused drugs mainly related to people's values, motivation, and societal demand, whereas barriers mainly encompassed practical issues, including knowledge, time, financial resources, and legal conditions. Strategies emphasizing the benefits of redispensing and further streamlining process compatibility could support implementation.

## Highlights

• The redispensing of unused oral anticancer drugs reduces waste, thereby providing economic and environmental benefits.
• This study identified barriers and facilitators to the implementation of redispensing unused oral anticancer drugs.
• Facilitators mainly related to societal benefits, whereas barriers mainly encompassed practical issues in the pharmacy.
• Implementation strategies should focus on emphasizing benefits and streamlining the redispensing process’ compatibility.

## 1 Introduction

Considerable quantities of medication go to waste after patients discontinue their treatment, for instance due to side effects or lack of effect.^1^, 2., 3 Patients are currently advised to dispose of unused medication through take-back programs in pharmacies to use prevent inappropriate of unused medication by others and to mitigate environmental pollution resulting from improper disposal methods (i.e., disposing in the household garbage or flushing down the toilet or sink).^4^, 5., 6 However, these take-back programs fall short in addressing the waste of economic and environmental resources linked to medication disposal. Simultaneously, increasing financial and environmental deficits give rise to concerns of sustainable medication use.^7^ Therefore, it is desirable to shift from mere waste-management to comprehensive waste-minimisation.^8^

Beyond collecting unused medication from patients, the pharmacy could redispense unused medication of good quality to other patients. Such redispensing programs are currently being carried out on a limited scale to help patients with lower incomes or those with limited access to medication.9., 10, 11, 12. For greater sustainability, redispensing should however be expanded to a larger scale, and thus implementation in clinical care is needed.^13^^,^^14^ Healthcare providers, patients, general public and other stakeholders are supportive of redispensing if medication quality is verified upon redispensing.15., 16., 17., 18, 19 This could be established through the use of temperature-monitoring devices and sealed packaging, ensuring medication's quality and authenticity.^20^, ^21^, ^22^ Due to the costs of temperature-monitoring devices and sealed packaging, as well as the additional workload for the pharmacy*,* redispensing is specifically suitable for expensive medications that frequently remain unused,^23^ like oral anticancer drugs (OADs^1^). A recent prospective trial (the ROAD-study) showed that for over 1000 patients redispensing unused OADs is feasible, offers substantial economic advantages^24^ and can benefit the environment.^25^

However, proving the effectiveness of an intervention does not guarantee its sustained implementation in clinical care as this requires behavioural and systemic changes.^26^ These changes can be either hindered or supported by various barriers and facilitators related to the innovation itself, the process and the context.^27^^,^^28^ Since the ROAD-study represents the first implementation and evaluation of a redispensing program in clinical care in four Dutch hospitals, findings can already inform broader implementation strategies.^29^^,^^30^ Furthermore, the experiences of people involved in the intervention could serve as a source for other settings interested in waste-minimization and contribute to the international dialogue on redispensing unused medication.31., 32, 33., 34., 35. The aim of this study was thus to identify barriers and facilitators to the implementation of redispensing unused OADs in clinical care.

## 2 Materials and methods

### 2.1 Design and setting

This study employed a hybrid effectiveness-implementation type I trial design,^29^ identifying barriers and facilitators to implementation during a prospective multicentre trial in the Netherlands (the ROAD-study).^24^ Semi-structured individual interviews were conducted with key stakeholders involved in the ROAD-study, including representatives of all four participating hospitals: two academic medical centers (Radboud University Medical Center, located in Nijmegen, and University Medical Center Utrecht, located in Utrecht) and two teaching hospitals (Jeroen Bosch Hospital, located in ‘s Hertogenbosch, and St. Antonius Hospital, located in Utrecht, Nieuwegein and Woerden). The study protocol was registered in the WHO International Clinical Trials Registry Platform (ICTRP) under the identifier NL9208.^36^ The Consolidated Criteria for Reporting Qualitative Research (COREQ) were followed to assure explicit and comprehensive reporting (Additional File 1).

### 2.2 The intervention

The ROAD-study and its redispensing procedure have been previously described.^24^ In summary, outpatient pharmacies provided all prescribed OAD packages in sealed bags equipped with a time-temperature indicator to study participants. Participants were instructed to return unused OAD packages to the pharmacy, where the quality was assessed based on three criteria: 1) enclosed seal and outer packaging; 2) a remaining shelf-life of at least six months and 3) storage temperature in accordance with the product label claim. If all quality criteria were met, the OAD package was *returned to the stock* to be redispensed to another study participant. In this case, the original payment was refunded.

### 2.3 Participants

To obtain a diverse sample of key stakeholders involved in redispensing unused OADs, we aimed to include at least five pharmacy employees (including at least one pharmacist, pharmacy technician and pharmacy assistant), five clinicians prescribing OADs (including at least one oncologist, haematologist, urologist and specialized oncology nurse), five patients who participated in the trial and five patients who declined trial participation. Various recruitment strategies were employed: 1) pharmacy employees and prescribing clinicians were invited by e-mail, using convenient and snowball sampling methods; 2) participating patients were recruited through one of the redispensing trial newsletters, and were chronologically selected; and 3) random sampling was used to recruit patients who declined trial participation treated in an academic center by post. All interviewees received written study information and provided informed consent verbally (pharmacy employees and prescribing clinicians) or in writing (patients) prior to the interview.

### 2.4 Conceptual framework

The Consolidated Framework for Implementation Research (CFIR) guided data collection and analysis. The CFIR is a well-established framework for assessing factors influencing implementation success.^28^ The updated CFIR consists of 48 constructs and 19 subconstructs,^28^ categorised in 5 domains: innovation (i.e., the innovation being implemented), outer setting (i.e., setting in which the inner setting exists), inner setting (i.e., the context in which the innovation is implemented), individuals (i.e., individual roles and individual characteristics), and the implementation process (i.e., the activities and strategies used to implement the innovation). Throughout this study, barriers and facilitators were systematically assessed using the CFIR, providing structured insights that can inform implementation strategies.

### 2.5 Interviews

The interview guide was based on CFIR domains relevant for the stakeholder group, which was assessed by the first and last author independently, and was thereafter discussed with all authors until consensus was reached. Questions were related to experiences during the trial, but also encompassed suggestions for future implementation. To prevent bias related to the ROAD-study research team's involvement, interviews were conducted by an independent interviewer. This interviewer, a master student in pharmacy with prior interviewing experience, had no role in the ROAD-study nor prior relationships with interviewees. Interviews were conducted halfway throughout the trial period, taking place between 11 and 15 months from the trial's start (January 2022 – June 2022). After the first two interviews, the interview guide and obtained data were evaluated and extensively discussed by the interviewer and first author. The final topic guide was developed iteratively, with adjustments made after the first two, six and eight interviews (Additional file 2).

Interviews were conducted in person, by telephone or via online videoconferencing using Microsoft Teams, depending on the preference of the interviewee. All interviews were audio recorded and field notes were taken. Data comprehensiveness and saturation was checked by the first author and discussed with the interviewer. Data saturation, defined as no new relevant information emerging from the data per target group, was reached and confirmed with two additional interviews per target group. Interviewees received their transcripts to check accuracy, but no abnormalities were encountered.

### 2.6 Data analysis

Interviews were transcribed verbatim and anonymised prior to analysis in Atlas.ti for Windows (Version 23.1.1). To familiarise with the data, all recordings were relistened prior to analysis. Thematic content analysis was used to report the meaning and content of patterns. To this end, inductive open and axial coding was conducted by the first author by labelling relevant text fragments with codes and grouping matching codes. Open codes of seven transcripts and all axial codes were checked by the second author and discrepancies were resolved by discussion. Subsequently, themes were defined by discussing interrelationships among the axial codes between the research team until consensus was reached. Also the deductive categorisation of themes into the CFIR framework was established through discussion with the research teamuntil consensus was reached. Quotes that illustrated the themes were selected and translated by the first two researchers, with translation assisted by an online translator (DeepL) before quotes were collaboratively reviewed and adjusted for readability.

## 3 Results

In total, 35 individuals involved in redispensing unused OADs were interviewed in person (*n* = 2), via Microsoft teams (*n* = 19) or via telephone (*n* = 14) (Additional File 3). The sample included ten pharmacy employees (twelve approached), seven prescribing clinicians (eleven approached), eleven patients participating in the trial (study advertisement in the newsletter distributed to 838 respondents resulted in 37 patients expressing interest), and seven patients who declined trial participation (68 approached). Five pharmacy employees and one oncologist that were interviewed were part of the local implementation team. One patient joined the interview with their partner. Invitees whom did not participate had a lack of time (two prescribing clinicians) or provided no response (two pharmacy employees, two prescribing clinicians and 49 patients who declined trial participation).

Interviews had a median duration of 25 min (range: 10–55 min), being the shortest for prescribing clinicians and patients who declined trial participation(both medians: 14 min), while interviews with pharmacy employees (median: 30 min) and participating patients (median: 34 min) had the longest duration.

**Fig. 1:**
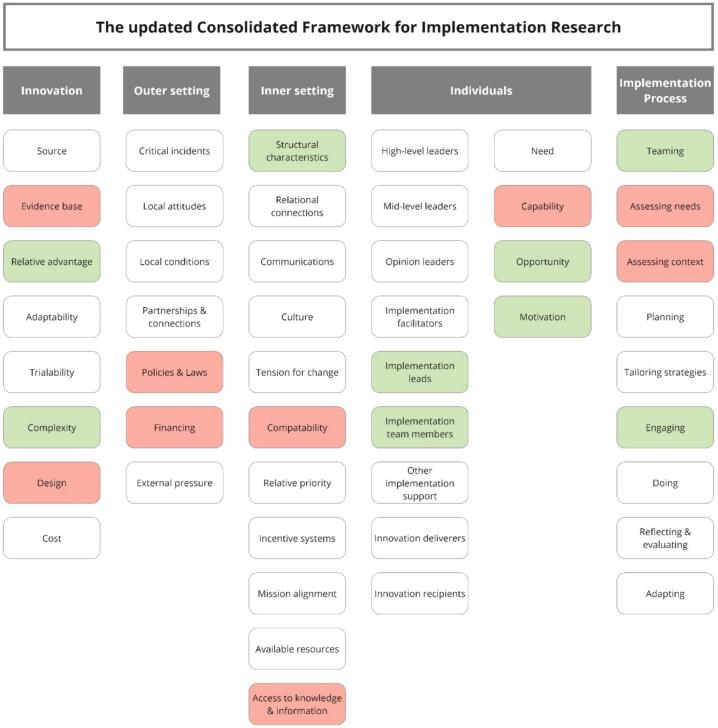
Overview of relevant Consolidated Framework for Implementation Research (CFIR) constructs relating to barriers (red) and facilitators (green) to the implementation of redispensing unused oral anticancer drugs. (For interpretation of the references to colour in this figure legend, the reader is referred to the web version of this article.)

Barriers and facilitators to the implementation of redispensing unused OADs encompassed all CFIR domains (Fig. 1). The outer setting only encompassed barriers, whereas only facilitators were identified in the domain roles of individuals. In a more comprehensive analysis, we subsequently identified 15 themes encompassing barriers and facilitators to the implementation of redispensing unused OADs in clinical care (Table 1).

**Table 1:** Barriers and facilitators to the implementation of redispensing unused oral anticancer drugs categorised to the updated Consolidated Framework for Implementation Research (CFIR).

Domain	CFIR Construct	B(arrier)/ (F)acilitator	Theme	Concerning
Innovation	Complexity, Relative advantage	F	Convenient process requiring an acceptable time-investment	Pharmacy employees & patients
	Design, Evidence Base	B	Unclear target population	Pharmacy employees & patients
Outer setting	Policies & Laws	B	Redispensing legally prohibited	Pharmacy employees
	Financing	B	Absence of financial compensation for pharmacies	Pharmacy employees
Inner setting	Compatibility (B), Structural characteristics (F)	B	Complexity arising from two parallel work processes	Pharmacy employees
		F		
	Access to knowledge & information	B	Widespread communication on adjustments within local teams challenging	Pharmacy employees
Individual roles	Implementation leads, Implementation team members	F	Support from project leaders and implementation champions	Pharmacy employees
Individual characteristics	Capability	B	Patient's low receptiveness due to the burden of oncology treatment	Patients
	Motivation	F	Being well-motivated by personal values and societal impact	All
	Opportunity	F	Feeling ensured of medication quality upon redispensing	All
Implementation process	Assessing needs, Assessing context	B	Lack of familiarization among pharmacy technicians	Pharmacy employees
	Engaging	F	Endorsement by healthcare providers for patient participation	All
		F	Clear and personal patient communication	Patients
		F	Good visibility of intervention's success	All
	Teaming	F	Implementation well supported through a collaborative network	Pharmacy employees

## 4 Innovation

### 4.1 Convenient process requiring an acceptable time-investment

Generally, procedures of redispensing unused OADs were perceived convenient and required an acceptable time-investment. Redispensing procedures executed by pharmacy technicians and assistants appeared most labour-intensive, particularly in the starting phase when the staff was not yet familiarized with the tasks, but pharmacy employees felt that they could manage it without compromising other tasks. It was, for instance, argued that no extra staff needed to be hired to support these tasks.

*“I was surprised that it [redispensing] hadn't been done before and I thought the reason was that it would be so much more comprehensive and much more work, while it's actually okay.” – Pharmacy employee.*

Patient participation consisted of the return of unused medication and temperature indicators, which was perceived as an acceptable time-investment. Most participants mentioned that this could be arranged during regular hospital visits, thus not requiring extra time.

*“It's of course a little extra work, you get e-mails to which you have to pay attention, and you have to return temperature indicators, but those things have no objection to me*.” *– Patient, trial participant.*

Prescribing clinicians felt that their role was limited to support, hence requiring limited effort. Both prescribing clinicians and pharmacy employees found redispensing more feasible than other waste-preventive measures from a workload standpoint (i.e., reducing prescription and dispensing quantities, thus dispensing more frequently).

### 4.2 Unclear target population

A barrier for some pharmacy technicians and patients was that the reason for implementing redispensing for OADs was unclear. They believed that the possibility of OAD waste was low, because OADs are generally dispensed in small quantities, either per medication package or per treatment cycle. Since these patients would directly start with the medication package, using additional packaging materials to enable redispensing did not make sense to them, causing confusion. It also pressured fidelity as pharmacy technicians were tempted to not provide additional packaging in these situations.

*“Often when patients pick up their medication, the doctor already approved starting the treatment cycle…. so the patient still considers that … a waste of packaging material, because … you know it is being opened today anyway”. – Pharmacy technician.*

On the other hand, some participants declared that OADs provided a perfect proof-of-concept for redispensing unused medication, which should now also be endorsed for other medications that are frequently wasted.

## 5 Outer setting

### 5.1 Redispensing legally prohibited

Current law was perceived as a barrier for the implementation of redispensing unused OADs in clinical care, because it prohibits redispensing outside study settings. Particularly the falsified medicine directive (FMD) on the European level was mentioned as a legal obstacle. Legislation is also needed to mandate a standardized quality assurance procedure, according to a few participants.

“*For me, it's important that it's supported by the government, because the drug law now still states that if something has left the pharmacy, it has to be destroyed on its return. The FMD supports that as well*”. *– Pharmacist.*

### 5.2 Absence of financial compensation for pharmacies

Lack of financial compensation for required redispensing activities performed by pharmacies also was a barrier expressed by pharmacy employees. It was argued that most pharmacies are willing to contribute to waste-minimization, but require reimbursement for additional materials and labour (although limited). Withholding compensation could even provoke fraud through double declarations, according to a few participants.

*“Pharmacies are busy, so I think if we want to make time for this, you need compensation…. just like our regular prescription line fee”. – Pharmacy technicians.*

## 6 Inner setting

### 6.1 Complexity arising from two parallel work processes

Patient participation in redispensing unused OADs was contingent on their consent, resulting in two competing parallel work processes in the pharmacy. This was perceived as a barrier by pharmacy employees. To distinguish between participants and non-participants, registration in the hospital and pharmacy information systems was required, which was perceived as labour-intensive and susceptible to mistakes (i.e., mix-ups or overlooking patients). Moreover, as a consequence, a double-stock (i.e., one containing new drug packages and one containing drug packages approved for redispensing) was derived. This was also perceived as confusing and easy to overlook. Using functional adaptation to align redispensing procedures with regular working processes (i.e., putting the additional materials on convenient spots in the work process) felt helpful to pharmacy employees in overcoming this challenge. Convenient spots included the front-office desk and/or places were medications were collected and checked to be dispensed.

*“It has to be clear to the technician that a patient is participating. That it is displayed very clearly per dispensing ‘pay attention: this patient participates, dispense according to the ROAD-study’. Those have been complicated adjustments that we really had to pay attention to”. – Pharmacist.*

### 6.2 Widespread communication on adjustments within local teams challenging

Although pharmacy employees were mostly positive, they reported that uptake of alterations during the study were sometimes difficult due to the widespread communication required. Mainly the large size of the pharmacy team necessitated that these changes were well communicated and executed within the team. To illustrate, the policy on returning time-temperature indicators for reuse changed during the ROAD-study. This was received by the local project leader but not communicated well to all pharmacy employees, causing misunderstandings when patients wanted to hand in indicators.

“*The only thing is that the reuse of temperature loggers was not communicated clearly from the beginning, so it wasn't done. That was an adjustment, well, that wasn't picked up as well in the implementation because of that*”. – Pharmacist.

## 7 Individuals

### 7.1 Support from project leaders and implementation champions

Having a local project leader and implementation champions facilitated implementation of redispensing unused OADs. Pharmacy employees relied on these individuals (i.e., pharmacists and pharmacy technicians), who provided a pragmatic work process, informal opportunities for questions and short feedback loops to quickly overcome any issues.

*“If you're going to implement this, you do need someone who is responsible locally, so you can ask questions if something is off or not working well. I think that is important”. – Pharmacy technician.*

### 7.2 Patient's low receptiveness due to burden of oncology treatment

The burden of their disease and treatment was a barrier to patients, because they experienced difficulties in receiving information about redispensing unused OADs. They felt little energy for matters such as reading study information. A few patients did not even notice receiving the information, because they were too preoccupied by their health state. Others mentioned that they felt every action was too much at the time of receiving the invitation.

*“Even if the text is clear, the phase of your illness clouds your perception. I did have that during my first treatment. That I read something a few times and that I had to repeat to myself ‘do I read it right?’, ‘do I understand it right?’, ‘okay, I've lost it again now’. So a day later I read it again. But not everyone will be able to do it that way”. –* Patient, trial participant.

Due to patients' health condition, engagement of caregivers was suggested as being helpful. Caregivers could help with reading the information and explaining the process. In addition, caregivers could return unused OADs to the pharmacy after a patient was deceased.

### 7.3 Being well-motivated by personal values and societal impact

The societal impact that could be achieved by redispensing unused OADs was described as a facilitator by all participants, primarily because this aligned with personal values. Experiences of medication waste stimulated patient participation, or in case of one patient, made them regret not participating in redispensing. Specifically, the costs associated with medication waste under rising healthcare costs drove patients. Redispensing unused OADs could also improve environmental sustainability, according to some participants. For these reasons, it was argued that countering waste provided a thankful feeling, stimulating pharmacy employees and prescribing clinicians to perform the required tasks.

*“I do enjoy that when you do indeed get to put it [drug packages] back in one of the drawers, so it gets redispensed again and just, that we're not wasting it…. The same feeling as when you would separate your garbage at home, it kind of feels like that”.* – Pharmacy employee.

### 7.4 Feeling ensured of medication quality upon redispensing

Given the severity of an oncologic disease, ensured drug quality and adequate information about this was an important facilitator underlying patients' participation. For pharmacy employees, prescribing clinicians and most patients, the current quality assurance provided enough trust in product quality. Nevertheless, for a few patients concerns about drug quality or incomprehensible information about the quality procedure was the reason for not participating in redispensing.

“*What happens now is that the medications I receive are all sealed, so indeed well-kept and taken care of. Only at the time I use them, they can be opened, by me. And also that there is a check with such a magnet [temperature logger], in terms of, yes, temperature and validity of the medications. I felt that that provided enough care thus participated with that”. –* Patient, trial participant.

*“The moment you read something about reused medication being quality controlled, yes fine but how do they do that? Does somebody look at it? Or is it put under a microscope somewhere in a I don't know what? I couldn't imagine that. And so partly because of that I said; then we're not going to do that*”. – Patient, declined trial participation.

## 8 Implementation process

### 8.1 Lack of familiarization among pharmacy technicians

Particularly at the start, some pharmacy technicians felt insecure in executing tasks for redispensing. It took pharmacy technicians a while to get familiarized with tasks required for redispensing as they only had to be executed occasionally, which was perceived as a barrier. Pharmacy technicians proposed that having more work instructions that take into account personal preferences (i.e., practicality, extensiveness and repetitiveness) could support faster familiarization.

*“So, most pharmacy technicians know what to do, but then some just don't feel confident enough, because they don't do it enough…”.*– Pharmacy techician.

### 8.2 Endorsement by healthcare providers for patient participation

Endorsement by healthcare providers facilitated patient participation in redispensing, according to interviewees of most hospitals.

*“If you really want to motivate people, the verbal information should be provided by those directly involved, for example your doctor, or your pharmacist, that's mainly what motivates them [patients] to participate. With written explanations you don't have a relationship and with your doctor or with a pharmacist you do. So I think that works better to win people over”.* – Patient, trial participant.

Redispensing unused OADs was seen as a pharmacy-led program, yet a supportive role of the oncologist/haematologist and specialized nurses was perceived as crucial. Particularly the trust between prescribing clinicians and patients was seen as helpful for having a conversation about reasons to participate. Some patients argued that their oncologist determines every step of their treatment, thus should be aware of their participation. Other patients were already convinced of their oncologist's support and rather focused on clinical conversations during their consults. Finally, it was argued that prescribing clinicians in primary care should also be engaged to prevent disposal of unused OADs through community pharmacies.

*“I think there does need to be some additional information provision. Not only at the pharmacy, but also definitely at the healthcare providers, like the medical specialists, nursing specialists, but also the oncology nurses on the ward. To really give information, but from a confidential atmosphere. Someone they see regularly, who they have good contact with and not just at the pharmacy where they see well, very often another person already”.* – Specialized oncology nurse.

### 8.3 Clear and personal patient communication

All participants agreed on the importance of clear and personal communication towards patients. Most participants perceived communication as facilitator. Primarily, the opportunity to ask questions and having a personal conservation about redispensing with either a clinician or pharmacist, was perceived as essential. Furthermore, patients recommended using different types of media to provide information and keep the written information as brief as possible given their health status.

*“If I'd only have received the sealed packaging with the accompanying patient instruction, I'd probably would have felt differently… Like okay, this is it. But… by the pharmacy addressing that I participated in the program and pointing out the information, you get the feeling that it's a little bit different than the normal collection of medications. So the communication provided more clarity”.* – Patient, trial participant.

### 8.4 Good visibility of intervention's success

All participants felt motivated by the regular updates on achieved successes of the ROAD-study, such as the number of enrolled patients and the cost-savings obtained. It made them feel proud and gave them the feeling that they were a part of a big accomplishment. It was also argued that it could support participation of more patients.

“*The mail was fine, it was clear and made me think: ‘ah, look, there has been quite a bit of cost-savings after all’ and I was also a bit proud of that, I thought, well, this is also due to my contribution. It stimulated me positively, because I thought: ‘ok, it is tracked counted, it is measured and it provides benefits’. So I was very satisfied with that*”. *–* Patient, trial participant.

### 8.5 Implementation well supported through a collaborative network

Having a collaborative network around redispensing unused OADs facilitated pharmacy employees as they could exchange procedures and brainstorm on implementation barriers, which was particularly appreciated at the start. It felt that by being part of a larger collaboration, a bigger effect could be achieved. Additionally, collaboration provided the opportunity for benchmarking, which was also perceived as a facilitator.

“*I found it very useful. … it was very nice to hear already what the other people's experiences were and to get information from others, to hear whether or not they had embedded it into the regular process and whether or not they used the forms and how they found out which patients you had missed etc*.”. – Pharmacist.

## 9 Discussion

In this study, we identified 15 themes encompassing barriers and facilitators to the implementation of redispensing unused OADs in clinical care, based on experiences and perspectives of key stakeholders involved in a hybrid effectiveness-implementation type I trial. Facilitators mostly originated from motivation, people's values, and societal demand. Emphasizing the added value of redispensing unused OADs could thus be leveraged to motivate individuals in (future) implementation. Barriers predominantly encompassed unpractical aspects, such as knowledge, time, financial resources, and legal conditions. Therefore, it is imperative to further streamline compatibility of the redispensing process, especially in pharmacies, making it as user-friendly as possible. Despite the challenges posed by these barriers, implementation of the intervention in the ROAD-study was perceived successful as it demonstrated substantial cost-savings and environmental benefits.^24^^,^^25^ It could thus be argued that the motivation described outweighed the practical obstacles encountered. In addition, this systematic assessment of barriers and facilitators may facilitate the optimization of strategies to implement the redispensing of unused OADs in clinical care.

Participants generally were positive about redispensing unused OADs due to the potential societal impact, which aligned with their personal values. This is in line with a body of literature that describes a growing momentum towards sustainable healthcare in general,^37^, ^38^, ^39^, 40. and medication waste reduction more specifically.^19^^,^^41^ A frequently perceived trade-off in these studies is that improved sustainability may compromise healthcare's quality.^37^, ^38^, ^39^, 40. Considering redispensing, risks of incorrect storage conditions in patients' homes or falsifications indeed gave rise to concerns regarding drug quality.15., 16., 17., 18, 19 The main reasons for patients to decline participation in the ROAD-study included not expecting to have leftover medication, feeling too ill, not being interested in participating in research and quality concerns.^24^ In this study, three main facilitators helped to overcome these doubts: feeling ensured of drug quality upon redispensing, endorsement by all healthcare providers and clear, personal patient communication. These facilitators align with the outcomes of a previous study on the willingness of patients living with cancer to participate in redispensing.^19^

The redispensing of unused OADs was generally seen as a convenient procedure, yet some barriers existed, mainly among pharmacy employees. Complications arose due to the presence of two parallel work processes, hindering compatibility. Internal support structures (i.e., project leaders and implementation champions) and external support structures (i.e., a collaborative network) were used as facilitators to overcome this issue, but it was argued that it would be more convenient to standardize redispensing for all patients. By making redispensing a routine procedure for all patients, inconveniences related to patient registration and maintaining double stocks could be eliminated. Pharmacies monitor medication quality in the redispensing program, including medication authenticity and proper storage, and have the professional expertise to make decisions on medication quality upon redispensing. Accordingly, redispensing could be applied to other medications. However, before redispensing could be standardized, barriers still need to be overcome concerning the outer-setting, including legal and financial barriers.

The identified barriers and facilitators could inform future implementation strategies through the CFIR-Expert Recommendations for Implementing Change (ERIC) Implementation Strategy Matching tool.^42^^,^^43^ Some of the suggested strategies from this tool were already used during the trial. For instance, educational meetings to overcome the barriers of communicating adjustments in large teams ‘(Access to knowledge & information) and doubts about the target population (Design, Evidence base). The implementation strategy from this study appeared to be a good match, but the strategy could be improved by using the findings from the interviews, such as more appropriate familiarization for pharmacy employees. Furthermore, some other suggested ERIC strategies were not yet used, including tailoring the strategy, mainly to address barriers related to a lack of compatibility. Specifically, this strategy could be used to better address local needs, for instance by providing recommendations on functional adaptation to better align redispensing procedures with regular working processes. In this way, future implementation processes could be informed by the current study, supplemented with local context analysis.

Recommendations for the implementation of a drug redispensing program as proposed in this study were already used to inform a clinical guideline on redispensing unused OADs in the Netherlands.^44^ Furthermore, on an international level, the findings of this study could re-open the debate on redispensing unused medication by showing experiences of all involved stakeholders, and providing knowledge on barriers and facilitators in the Netherlands. Finally, findings could be extrapolated to the redispensing of other drug groups, because redispensing unused medication appeared cost-beneficial for medication packages exceeding a price of €100^23^ and can reduce environmental burdens of medication use.^25^

### 9.1 Strengths and limitations

The strength of this study lies in its ability to offer guidance on the implementation of redispensing unused OADs in clinical care based on the experiences obtained in this trial. By using a hybrid type 1 design, implementation recommendations were provided in an early stage of innovation development, accelerating the intervention's implementation. Moreover, identified barriers and facilitators were derived from all key stakeholders involved in the redispensing program, including patients who declined participation in redispensing, who were previously underrepresented.^19^

The study was, however, prone to some limitations. Firstly, implementation was evaluated in a trial setting, which may differ from implementation in clinical care, for instance due to the availability of trial support. A researcher coordinated implementation, performed frequent fidelity checks and intervened when needed. This was perceived as helpful by most participants, but may be too resource-intensive in a real-world setting.^26^ Secondly, interviewees were informed that data was analysed by this researcher, which may have caused reluctance to be critical despite the independent interviewer that was hired to create an open and safe atmosphere. Thirdly, one may speculate that the implementation strategy employed in the study was already well-crafted as it was perceived successful, though further details are needed to ascertain this. Finally, generalizability is a concern due to context-specific experiences. A diverse sample, including all involved stakeholders in four hospitals, was selected to minimize this bias. Still, extrapolation to other settings and countries must be done carefully, taking into account differences in healthcare systems, legal restrictions and payment systems. It must for instance be noted that the Dutch healthcare system features compulsory healthcare insurance ensuring that medications are universally accessible at no extra costs to patients, thus patients do not benefit financially from participating in redispensing. Despite these limitations, the ROAD-study still provided valuable information about implementation of redispensing of unused OADs.^29^

## 10 Conclusion

This study identified barriers and facilitators to the implementation of redispensing unused OADs in clinical care. Facilitators, were mainly associated with people's values, motivation, and societal demand, which outweighed barriers that predominantly encompassed practical issues, including knowledge, time, financial resources, and legal conditions. To optimize implementation, future strategies could emphasize the added value of waste-minimization and further streamline the redispensing process to better match space and staff resources. In conclusion, the understanding of these barriers and facilitators provides a foundation for the sustainable implementation of redispensing unused OADs in clinical care.

## Ethics approval and consent to participate

The study protocol was reviewed by the Medical Research Ethics Committee (METC Oost Nederland, reference 2020–7049) and the need for approval was waived.

## Consent for publication

Not applicable.

## Funding

This work was supported by the 10.13039/501100001826Netherlands organisation for health research and development (ZonMw, program rational pharmacotherapy, grant number: 848018008).

## CRediT authorship contribution statement

**Elisabeth M. Smale:** Writing – original draft, Visualization, Validation, Project administration, Methodology, Investigation, Formal analysis, Data curation, Conceptualization. **Eva W. Verkerk:** Writing – review & editing, Methodology, Formal analysis, Conceptualization. **Eibert R. Heerdink:** Writing – review & editing, Supervision, Methodology, Conceptualization. **Toine C.G. Egberts:** Writing – review & editing, Supervision, Methodology, Conceptualization. **Bart J.F. van den Bemt:** Writing – review & editing, Supervision, Methodology, Funding acquisition, Conceptualization. **Charlotte L. Bekker:** Writing – review & editing, Supervision, Methodology, Funding acquisition, Conceptualization.

## Declaration of competing interest

The authors declare that they have no competing interests.

## Data Availability

The datasets used and/or analysed during the current study are available from the corresponding author on reasonable request.

## Appendix A Supplementary data

The name for supplementary data is: Additional Files 1 - 3 Image 1

## Footnotes

1 OADs: Oral Anticancer Drugs
