# Supplementary material for: Barriers and facilitators to implement the redispensing of unused oral anticancer drugs in clinical care: A hybrid-effectiveness type I study
Source: Explor Res Clin Soc Pharm. 2024 Aug 17;15:100493. doi: 10.1016/j.rcsop.2024.100493 (PMC11388756; doi:10.1016/j.rcsop.2024.100493)
Supplement: Supplementary file 1 — The name for supplementary data is: Additional Files 1 - 3 [file mmc1.docx]

**Additional Files**

**Additional File 1** ‘COREQ (COnsolidated criteria for REporting Qualitative research) Checklist’ P2 – P3

**Additional File 2** ‘Interview guide’ P4 – P11

**Additional File 3** ‘Participant characteristics’ P12

**Additional File 1**

**COREQ (COnsolidated criteria for REporting Qualitative research) Checklist**

A checklist of items that should be included in reports of qualitative research. You must report the page number in your manuscript where you consider each of the items listed in this checklist. If you have not included this information, either revise your manuscript accordingly before submitting or note N/A.

| **Topic** | **Item No.** | **Guide Questions/Description** | **Reported in (section)** |
| --- | --- | --- | --- |
| **Domain 1: Research team and reflexivity** | | |  |
| *Personal characteristics* |  |  |  |
| Interviewer/facilitator | 1 | Which author/s conducted the interview or focus group? | Interviews |
| Credentials | 2 | What were the researcher’s credentials? E.g. PhD, MD | Interviews |
| Occupation | 3 | What was their occupation at the time of the study? | Interviews |
| Gender | 4 | Was the researcher male or female? | Interviews |
| Experience and training | 5 | What experience or training did the researcher have? | Interviews |
| *Relationship with participants* | | |  |
| Relationship established | 6 | Was a relationship established prior to study commencement? | Interviews |
| Participant knowledge of the interviewer | 7 | What did the participants know about the researcher? e.g. personal goals, reasons for doing the research | Interviews |
| Interviewer characteristics | 8 | What characteristics were reported about the interviewer/facilitator? e.g. Bias, assumptions, reasons and interests in the research topic | Interviews |
| **Domain 2: Study design** |  |  |  |
| *Theoretical framework* |  |  |  |
| Methodological orientation and Theory | 9 | What methodological orientation was stated to underpin the study? e.g.  grounded theory, discourse analysis, ethnography, phenomenology, content analysis | Conceptual framework |
| *Participant selection* |  |  |  |
| Sampling | 10 | How were participants selected? e.g. purposive, convenience, consecutive, snowball | Participants |
| Method of approach | 11 | How were participants approached? e.g. face-to-face, telephone, mail, email | Results |
| Sample size | 12 | How many participants were in the study? | Results |
| Non-participation | 13 | How many people refused to participate or dropped out? Reasons? | Results |
| *Setting* |  |  |  |
| Setting of data collection | 14 | Where was the data collected? e.g. home, clinic, workplace | Interviews / results |
| Presence of nonparticipants | 15 | Was anyone else present besides the participants and researchers? | Results |
| Description of sample | 16 | What are the important characteristics of the sample? e.g. demographic data, date | Results |

| **Topic** | **Item No.** | **Guide Questions/Description** | **Reported in (section)** |
| --- | --- | --- | --- |
| *Data collection* | | | |
| Interview guide | 17 | Were questions, prompts, guides provided by the authors? Was it pilot tested? | Interviews  Additional File 2 |
| Repeat interviews | 18 | Were repeat interviews carried out? If yes, how many? | N/A |
| Audio/visual recording | 19 | Did the research use audio or visual recording to collect the data? | Interviews |
| Field notes | 20 | Were field notes made during and/or after the interview or focus group? | Interviews |
| Duration | 21 | What was the duration of the interviews or focus group? | Results |
| Data saturation | 22 | Was data saturation discussed? | Interviews |
| Transcripts returned | 23 | Were transcripts returned to participants for comment and/or correction? | Interviews |
| **Domain 3: analysis and findings** | | |  |
| *Data analysis* |  |  |  |
| Number of data coders | 24 | How many data coders coded the data? | Data analysis |
| Description of the coding tree | 25 | Did authors provide a description of the coding tree? | Additional File 3 |
| Derivation of themes | 26 | Were themes identified in advance or derived from the data? | Data analysis |
| Software | 27 | What software, if applicable, was used to manage the data? | Data analysis |
| Participant checking | 28 | Did participants provide feedback on the findings? | Interviews |
| *Reporting* |  |  |  |
| Quotations presented | 29 | Were participant quotations presented to illustrate the themes/findings?  Was each quotation identified? e.g. participant number | Results |
| Data and findings consistent | 30 | Was there consistency between the data presented and the findings? | Results |
| Clarity of major themes | 31 | Were major themes clearly presented in the findings? | Results |
| Clarity of minor themes | 32 | Is there a description of diverse cases or discussion of minor themes? | Results |

Developed from: Tong A, Sainsbury P, Craig J. Consolidated criteria for reporting qualitative research (COREQ): a 32-item checklist for interviews and focus groups. *International Journal for Quality in Health Care*. 2007. Volume 19, Number 6: pp. 349 – 357

**Once you have completed this checklist, please save a copy and upload it as part of your submission. DO NOT** **include this checklist as part of the main manuscript document. It must be uploaded as a separate file.**

**Additional File 2**

## **Interview guide**

*Research question*

What are the barriers and facilitators to implementation of redispensing unused OADs in clinical practice?

*Framework*

Based on Consolidation Framework for Implementation Research (CFIR) framework.

*Overview of CFIR domains represented in the topic guide*

| *CFIR domain* | | *Providers* | *Patients* |
| --- | --- | --- | --- |
| Characteristics of individuals | Knowledge & Beliefs about the Intervention | X | X |
|  | Attributes | X | X |
|  | Self-efficacy | X | X |
| Intervention characteristics | Evidence strength and quality | X | - |
|  | Relative advantage | X | X |
|  | Adaptability | X |  |
|  | Trialability | X |  |
|  | Complexity | X | X |
|  | Design quality and packaging | X | X |
|  | Costs | X | X |
| Inner setting | Structural Characteristics | X | - |
|  | Culture | X | - |
|  | Tension for Change | X | X |
|  | Compatibility | X | - |
|  | Leadership Engagement | X | - |
|  | Available Resources | X | X |
|  | Access to Knowledge & Information | X | X |
| Outer setting | Patient Needs & Resources | X | X |
|  | Cosmopolitanism | X | - |
| Process | Planning | X | - |
|  | Executing | X | - |
|  | Available resources | X | - |
|  | Opinion leaders | X | - |
|  | Intervention participants | - | X |
|  | External Change Agents | X | - |

*Language*

The original topic guide was in Dutch as this is the native language of participants. For publication, the topic guide is translated into English. Translation was performed with DeepL (free version), an online Translating tool. Subsequently, translations were checked on accuracy using a backward-forward method by the first author (ES) and, if needed, adjusted to improve readability. The Dutch topic guide is available upon request.

***PATIENTS WHO DECLINED PARTICIPATION IN THE ROAD-STUDY***

*[Introduction of interviewer] *name**, master student in pharmacy, independent interviewer who is not involved in the redispensing program

Last year we invited you to participate in the redispensing of unused anticancer drugs. This involves patients returning unused drugs to the outpatient pharmacy and, if the drugs are of good quality, they could be used for another patient. You then decided not to participate.

Today I would like to hear your opinion about the redispensing program and your experience with the invitation to participate. We will use this information to help us improve the redispensing program. The interview will last approximately 30 minutes. The results of this interview will be processed anonymously for a scientific article and will be used to actually improve the program. Data will not be linked to your doctor nor to your pharmacy. This conversation will not affect your treatment pathway in any way.

Do you consent with recording the audio of this conversation? This will allow us to better study the conversation at a later time so that no valuable information is lost. The content of this conversation will remain confidential. The audio recording will be stored digitally and only researchers will have access to it. After the interview is transcribed, the audio recording will be deleted.

*[IF: written consent was obtained and the interviewee consents]* *Turn on sound recording*

| *Characteristics of individuals /* Knowledge and Beliefs about the Intervention & Self-efficacy & attributes |
| --- |

**How do you feel about redispensing unused anticancer drugs being offered at your hospital?**

**How did you experience the invitation to participate in the redispensing program? Why?**

- What kept you from participating in the redispensing program? Why?

Could you further elaborate on that?

- - Did particular activities, such as the return of unused medications, factor into your consideration?
    - If yes, what were these? What did you encounter? How can we accommodate you in this?
  - Did you experience any concerns about participating in the redispensing program?
    - If yes, what were these? What did you encounter? How can we accommodate you in this?

**Did you also perceived benefits to participating in the redispensing program?**

- If yes, could you elaborate on these?

| *Outer setting /* Patient Needs & Resources |
| --- |

**What would you need to potentially participate in the redispensing program in the future?**

| *Process /* Intervention participants |
| --- |

**How could patients be supported to participate in the redispensing program?**

| *End* |
| --- |

**Is there anything else related to drug waste or redispensing unused drugs that you would like to discuss?**

***PATIENT (ROAD-STUDY PARTICIPANT)***

*[Introduction of interviewer] *name**, master student in pharmacy, independent interviewer who is not involved in the redispensing program

Last year we invited you to participate in the redispensing of unused anticancer drugs. This involves patients returning unused drugs to the outpatient pharmacy and, if the drugs are of good quality, they could be used for another patient. You then decided not to participate.

Today I would like to hear your opinion about the redispensing program and your experience with the invitation to participate. We will use this information to help us improve the redispensing program. The interview will last approximately 30 minutes. The results of this interview will be processed anonymously for a scientific article and will be used to actually improve the program. Data will not be linked to your doctor nor to your pharmacy. This conversation will not affect your treatment pathway in any way.

Do you consent with recording the audio of this conversation? This will allow us to better study the conversation at a later time so that no valuable information is lost. The content of this conversation will remain confidential. The audio recording will be stored digitally and only researchers will have access to it. After the interview is transcribed, the audio recording will be deleted.

*[IF: written consent was obtained and the interviewee consents]* *Turn on sound recording*

| *Characteristics of individuals /* Knowledge and Beliefs about the Intervention & Self-efficacy & attributes |
| --- |

**How do you feel about redispensing unused anticancer drugs being offered at your hospital?**

**Do you think that redispensing unused drugs could counter medication waste? Can you elaborate on that?**

- Why do you think that?
- To what extent did you encounter this during your participation? If so, could you please share your experiences?

**How did you experience participation in the redispensing program? Why?**

- What motivated you to participate in the redispensing program? Why?
- Did you encounter any problems or barriers during the redispensing program? What were they and how did you deal with this?
  - Were there times or situations when you found participation difficult? If yes, which ones? Why?
    - Special activities? (e.g. return medication). If yes, which ones were these? What did you encounter?
    - What skills do you need to be able to participate in redispensing? Were you able to do this?
  - Did you experience any (other) concerns about participating in the redispensing program?
    - If yes, what did you encounter?
    - How did you deal with them?

| *Intervention characteristics / C*omplexity, Adaptability, Design quality and packaging & Costs  *Process / Access to Knowledge & Information* |
| --- |

- - **Did you perceive any aspect(s) of the redispensing program unclear or inconvenient?**
    - If yes, what did you encounter? What helped you to overcome this issue?

**I would now like to elaborate with you on some specific aspects of the redispensing program.**

Information provision (incl supporting materials, such as flyers):

- What did you think of the information provision (incl supporting materials, such as flyers)?
  - What did you like?
  - Did you miss anything? How could we accommodate you in this?
- What should the information provision look like in the future?
- What information would you like to receive?
- How do you want to be informed?
- Who should inform you?

Communication with your physician about redispensing unused drugs:

- What did you think of the communication with your physician?
  - What did you like?
  - Did you miss anything? How could we accommodate you in this?
- What should the communication look like in the future?
- What information would you like to receive from your physician?
- How and how often would you like to be informed by your physcian?

Communication with your pharmacy about redispensing unused drugs:

- What did you think of the communication with your pharmacy?
  - What did you like?
  - Did you miss anything? How could we accommodate you in this?
- What should the communication look like in the future?
- What information would you like to receive from your pharmacy?
- How and how often would you like to be informed by your pharmacy?

Support of the hospital:

- What did you think of the support provided by your hospital to participate in the redispensing program? vond u van de ondersteuning vanuit uw ziekenhuis om deel te nemen aan heruitgifte?
- What did you like?
- Did you miss anything? How could we accommodate you in this?

Reward/Thank you gift

- To what extent did you expect something in return for participating in the redispensing program? E.g., a reward or thank you?
  - To what extent could this improve your adherence to the instructions?
- Other:
- Did you miss anything else? How could we accommodate you in this?
- Are there any aspects that you think that should remain unaltered? Could you share your experiences with these aspects?
- **Did you experience any additional costs associated with participating in the redispensing program, such as expenses related to returning medication?**
  - How acceptable do you find incurring these costs for participation in a redispensing program?

| *Inner setting* / Tension for change |
| --- |

**How do you feel about the idea of extending the redispensing of unused anticancer drugs to all hospitals in the Netherlands?**

| *Outer setting /* Patient Needs & Resources |
| --- |

- What barriers currently exist for patients to participate in redispensing?
- How can we assist patients in overcoming these barriers?

| *Process /* Intervention participants |
| --- |

How can we best motivate patients to participate in the redispensing program?

| *Intervention characteristics / Relative advantage* |
| --- |

**Now that we've discussed redispensing unused drugs, are there any other strategies you can think of to minimize the waste of anticancer drugs, such as individualized dispensing strategies?**

- If so, which strategy do you favor, and what are your reasons for that preference?

| *End* |
| --- |

**Is there anything else related to drug waste or redispensing unused drugs that you would like to discuss?**

***PHARMACY EMPLOYEES***

*[Introduction of interviewer] *name**, master student in pharmacy, independent interviewer who is not involved in the redispensing program

Last year we invited you to participate in the redispensing of unused anticancer drugs. This involves patients returning unused drugs to the outpatient pharmacy and, if the drugs are of good quality, they could be used for another patient. You then decided not to participate.

Today I would like to hear your opinion about the redispensing program and your experience with the invitation to participate. We will use this information to help us improve the redispensing program. The interview will last approximately 30 minutes. The results of this interview will be processed anonymously for a scientific article and will be used to actually improve the program. Data will not be linked to your doctor nor to your pharmacy. This conversation will not affect your treatment pathway in any way.

Do you consent with recording the audio of this conversation? This will allow us to better study the conversation at a later time so that no valuable information is lost. The content of this conversation will remain confidential. The audio recording will be stored digitally and only researchers will have access to it. After the interview is transcribed, the audio recording will be deleted.

*[IF: interviewee orally consents]* *Turn on sound recording*

| *Characteristics of individuals /* Knowledge and Beliefs about the Intervention & Self-efficacy & attributes |
| --- |

**How do you feel about redispensing unused anticancer drugs being offered at your hospital?**

**Do you think that redispensing unused drugs could counter medication waste? Can you elaborate on that?**

- Why do you think that?
- To what extent did you encounter this during your participation? If so, could you please share your experiences?

**How did you experience working in the redispensing program? Why?**

**What motivated you to conduct the activities required for the redispensing program? Why?**

**Did you encounter any problems or barriers during the redispensing program? What were they and how did you deal with this?**

- - Were there times or situations when you found participation difficult? If yes, which ones? Why?
    - Special activities? (e.g. return medication). If yes, which ones were these? What did you encounter?
  - Did you experience any (other) concerns about participating in the redispensing program?
    - If yes, what did you encounter?
    - How did you deal with them?

| *Intervention characteristics / C*omplexity |
| --- |

**Did you perceive any aspect(s) of the redispensing program unclear or inconvenient?**

- - If yes, what did you encounter? What helped you to overcome this issue/these issues?

**To what extent does the redispensing program aligns with the existing work processes in your hospital? What adjustments have been made? (e.g., work processes, ICT). Were these changes manageable?**

- For instance, did the program require additional time?
- Was there a need for extra space to store materials?
- How were your colleagues instructed, and was the guidance provided clear?

| *Inner setting* / Structural characteristics, Compatibility, Leadership Engagement |
| --- |

**In the following section, I would like to focus on the implementation of the redispensing program for unused anticancer medications within your hospital.**

- How did you perceive the support for initiating the redispensing program among your colleagues? How did this support, or the lack thereof, affect you personally?
- How did your organization's team leaders or management respond to it? How did their response impact you?

| *Process* / Planning, Executing |
| --- |

**How did the implementation of the redispensing program for unused anticancer medications progress within your hospital?**

- What challenges did you encounter during this process, and what factors or resources were particularly helpful to you? [For team members only]

| *Outer setting* / Cosmopolitanism |
| --- |

- To what extent did you find it useful and important to exchange approaches with different hospitals through group meetings with other participating centers?
  - Do you consider this something that should be continued?
  - What information that you gained or shared during these meetings was the most valuable to you?
  - What information do you believe was still missing from your perspective?

| *Intervention characteristics /* Adaptability, Design quality and packaging, Costs |
| --- |

**I would now like to elaborate with you on some specific aspects of the redispensing program.**

- What did you think of the instructions regarding the implementation of redispensing? (e.g., presentations, guidelines, and newsletters)
  - What did you like?
  - Did you miss anything? How could we accommodate you in this?
  - What should the information provision look like in the future?
- What are your thoughts on the dispensing, return opportunities, quality assurance procedure and redispensing?
  - What did you like? Also consider logistics and administration.
  - Did you miss anything? How could we accommodate you in this?
  - Do you have any recommendations for the future?
- How do you feel about the additional costs made for the redispensing program? Also consider costs of materials (i.e., sealbags and time-temperature indicators) and work procedures in the pharmacy?
  - How acceptable do you find these costs?
  - How could structural reimbursement be established for scale-up as regular care?
- Were there any elements missing during the implementation of redispensing? How could we assist you further in this regard?

| *Inner setting* Tension for change |
| --- |

**We have the ambition to scale-up the redispensing of unused anticancer drugs to all hospitals in the Netherlands.**

**How do you feel about this?**

- What is your perspective on this? (i.e., added value?)
- What medications should this be applied to?

| *Intervention Characteristics* / Evidence Strength and Quality & Trialability |
| --- |

- **Do you believe there is sufficient evidence to implement redispensing and involve other centers?**
  - What information do you think is still missing?

| *Process* / Opinion Leaders & External Change Agents |
| --- |

**Based on your experiences, could you perhaps outline who you believe are necessary for implementing redispensing within a hospital?**

- Within the organization? (leadership, pharmacists, physicians, managers, Board of Directors, etc.)
- Outside your organization?

| *Intervention characteristics / Relative advantage* |
| --- |

**Now that we've discussed redispensing unused drugs, are there any other strategies you can think of to minimize the waste of anticancer drugs, such as individualized dispensing strategies?**

- If so, which strategy do you favor, and what are your reasons for that preference?

| *End* |
| --- |

**Is there anything else related to drug waste or redispensing unused drugs that you would like to discuss?**

***Physicians***

*[Introduction of interviewer] *name**, master student in pharmacy, independent interviewer who is not involved in the redispensing program

Last year we invited you to participate in the redispensing of unused anticancer drugs. This involves patients returning unused drugs to the outpatient pharmacy and, if the drugs are of good quality, they could be used for another patient. You then decided not to participate.

Today I would like to hear your opinion about the redispensing program and your experience with the invitation to participate. We will use this information to help us improve the redispensing program. The interview will last approximately 30 minutes. The results of this interview will be processed anonymously for a scientific article and will be used to actually improve the program. Data will not be linked to your doctor nor to your pharmacy. This conversation will not affect your treatment pathway in any way.

Do you consent with recording the audio of this conversation? This will allow us to better study the conversation at a later time so that no valuable information is lost. The content of this conversation will remain confidential. The audio recording will be stored digitally and only researchers will have access to it. After the interview is transcribed, the audio recording will be deleted.

*[IF: interviewee orally consents]* *Turn on sound recording*

| *Characteristics of individuals /* Knowledge and Beliefs about the Intervention & Self-efficacy & Attributes |
| --- |

**How do you feel about redispensing unused anticancer drugs being offered at your hospital?**

**Do you think that redispensing unused drugs could counter medication waste? Can you elaborate on that?**

- Why do you think that?
- To what extent did you encounter this during your participation? If so, could you please share your experiences?

| *Outer setting* / Patient Needs & Resources |
| --- |

**How did you feel about informing patients about redispensing?**

- How did your patients respond to this information, and how did it affect you?

| *Intervention characteristics / C*omplexity |
| --- |

**Did you perceive any aspect(s) of the redispensing program unclear or inconvenient?**

- If yes, what did you encounter? What helped you to overcome this issue/these issues?

Did you have any concerns regarding the redispensing program? If so, what were they, and how did you address them?

| *Inner setting* / Structural characteristics, Compatibility, Leadership Engagement |
| --- |

**In the following section, I would like to focus on the implementation of the redispensing program for unused anticancer medications within your hospital.**

- How did you perceive the support for initiating the redispensing program among your colleagues? How did this support, or the lack thereof, affect you personally?
- How did your organization's team leaders or management respond to it? How did their response impact you?

| *Intervention characteristics / Relative advantage* |
| --- |

**Now that we've discussed redispensing unused drugs, are there any other strategies you can think of to minimize the waste of anticancer drugs, such as individualized dispensing strategies?**

- If so, which strategy do you favor, and what are your reasons for that preference?

| *End* |
| --- |

**Is there anything else related to drug waste or redispensing unused drugs that you would like to discuss?**

=

**Additional File 3**

## **Participant characteristics**

**Table 1: Participant characteristics**

| **Participant** | **Role** | **Occupation** | **Center** | **Implementation team?** |
| --- | --- | --- | --- | --- |
| P1 | Pharmacy | Technician | A1 | Yes |
| P2 | Pharmacy | Technician | A1 | No |
| P3 | Pharmacy | Assistant | T1 | No |
| P4 | Pharmacy | Pharmacist | T1 | Yes |
| P5 | Pharmacy | Technician | T1 | No |
| P6 | Pharmacy | Pharmacist | T2 | Yes |
| P7 | Pharmacy | Technician | T2 | No |
| P8 | Pharmacy | Pharmacist | A2 | Yes |
| P9 | Pharmacy | Technician | A2 | No |
| P10 | Pharmacy | Technician | A2 | Yes |
| P11 | Clinician | Specialized nurse urology | A1 | No |
| P12 | Clinician | Oncologist | A1 | Yes |
| P13 | Clinician | Specialized nurse oncology | T1 | No |
| P14 | Clinician | Specialized nurse haematology | T2 | No |
| P15 | Clinician | Haematologist | T2 | No |
| P16 | Clinician | Junior oncologist | A2 | No |
| P17 | Clinician | Haematologist | A2 | No |
| P18 | Patient | Trial participant | A1 | No |
| P19 | Patient | Trial participant | A1 | No |
| P20 | Patient | Trial participant | T1 | No |
| P21 | Patient | Trial participant and partner | T1 | No |
| P22 | Patient | Trial participant | T1 | No |
| P23 | Patient | Trial participant | T2 | No |
| P24 | Patient | Trial participant | T2 | No |
| P25 | Patient | Trial participant | A2 | No |
| P26 | Patient | Trial participant | A2 | No |
| P27 | Patient | Trial participant | A2 | No |
| P28 | Patient | Trial participant | A2 | No |
| P29 | Patient | Declined trial participation | A1 | No |
| P30 | Patient | Declined trial participation | A1 | No |
| P31 | Patient | Declined trial participation | A1 | No |
| P32 | Patient | Declined trial participation | A1 | No |
| P33 | Patient | Declined trial participation | A2 | No |
| P34 | Patient | Declined trial participation | A2 | No |
| P35 | Patient | Declined trial participation | A2 | No |

A = Academic Center; T = Teaching center
